# Years After a Fire, Biocrust Microbial Communities are Similar to Unburned Communities in a Coastal Grassland

**DOI:** 10.1007/s00248-022-02137-y

**Published:** 2022-11-08

**Authors:** Brianne Palmer, Dawn Lawson, David A. Lipson

**Affiliations:** 1 Department of Biology, San Diego State University, San Diego, CA USA; 2 Department of Plant Science, University of California, Davis, Davis, CA USA

**Keywords:** Fire, Biocrust, Restoration, Grasslands

## Abstract

Microbial communities are integral for ecosystem processes and their taxonomic composition and function may be altered by a disturbance such as fire. Biocrusts are composed of macroscopic and microscopic organisms and are important for a variety of ecosystem functions, such as nutrient cycling and erosion control. We sought to understand if biocrust community composition and function were altered 1 year after a prescribed fire and 6 years after a wildfire in a coastal California grassland on San Clemente Island. We used shotgun metagenomic sequencing and measurements of chlorophyll content, exopolysaccharide production related to soil stability, and nitrogen fixation. There were no differences in the community composition between unburned samples and the samples burned in the prescribed fire and wildfire. Chlorophyll content differed between the prescribed fire and the controls; however, there were no measured differences in exopolysaccharide production, and nitrogen fixation. However, the wildfire and their respective unburned samples had different functions based on the gene annotations. We compiled one Actinobacteria metagenome-assembled genome from the shotgun sequences which had genes for oxidative and heat stress tolerance. These results suggest that the biocrust community can reach a community composition and function similar to the unburned biocrusts within a year after a prescribed burn and 6 years after a wildfire. However, legacy effects of the wildfire may present themselves in the differences between functional gene sequences. Due to their ability to match the undisturbed community composition and function within years and without intervention, future restoration work should consider the biocrusts in their restoration plans as they may provide valuable ecosystem functions after a disturbance.

**Supplementary Information:**

The online version contains supplementary material available at 10.1007/s00248-022-02137-y.

## Introduction

Microbial communities are an integral part of ecosystems worldwide with important influences on ecosystem and community structure and function. Because they are cryptic and difficult to study, our understanding of their contributions is limited, though growing advances in methodology have reduced costs and made next-generation sequencing methods more accessible, allowing us to apply sequencing methods to understand the complex interactions between microbial communities and their environment.

Wildland fire regimes are an important driver of ecosystem processes and are changing due to climate change [1, 2]. These changes have important implications for ecosystem dynamics and community structure and function [2]. Improving our understanding of microbial community response to fire will help us predict the ecosystem effects of climate change and develop ecosystem management strategies.

Fire can change soil nutrient inputs, soil temperature, and soil moisture [3]. Fire can also directly kill soil microbes by heating the soil surface [3]. Microbial groups differ in their fire tolerance, with fungi being the most sensitive to heat from a fire followed by nitrite oxidizers, and other heterotrophic bacteria [4].

Fire can alter the microbial community composition and function by changing the taxa and the functional genes [5–8] present, where the magnitude of the effect depends on the severity of the fire [9]. The effects of fire can be seen immediately after a burn and may result in an increase in *Acidiobacteria*, *Proteobacteria*, *Actinobacteria*, and *Firmicutes* [6–8]. For instance, in a Mediterranean ecosystem in Spain, soils burned in a wildfire had a higher diversity of Bacteria and Archaea compared to unburned fires [8]. Fire can also alter the soil properties and change microhabitats, thereby changing the microbial community composition [10, 11]. A metagenomic analysis of burned rhizosphere soil showed an increase in the number of unique nitrogen fixation genes and a reduction in the number of denitrification gene copies after a fire [12]. The amount of time since fire is an important factor in considering the changes in the microbial community. Community composition changes immediately after the fire [7] and it may take several years for the community structure to become similar to undisturbed sites [13]. For example, in a sagebrush-grassland ecosystem, microbial communities were determined to be similar to the mature soil 7 years after a fire [13]. Often, it is the top few centimeters of the soil surface that are most severely impacted by fire [14].

Biocrusts are communities of microorganisms, lichens, and bryophytes bound to the soil surface and are therefore directly exposed to fires [15]. These communities are important for soil stability, nitrogen fixation, and photosynthesis [16, 17]. After an ecological disturbance, recovery of biocrust communities can take anywhere from less than a year to many centuries [18], depending on the type of disturbance. In particular, it is estimated that it takes biocrusts less than 5 to 15 years to recover after fire, often depending on the biocrust type [19–21]. After a disturbance, Cyanobacteria are generally the first organisms to recolonize, followed by larger organisms such as lichens and bryophytes [22]. Biocrust communities differ in their taxa and function depending on the biocrust type or successional stage [23]. A comparative metagenomic study in from the Tengger Desert, China, found that *Actinobacteria* was the most abundant phylum in both early and late successional biocrusts but the biocrust types differed in the abundance of other common phyla—*Proteobacteria*, *Acidobacteria*, *Cyanobacteria*, *Planctomycetes*, and *Bacteroidetes* [23]. The genera *Rubrobacter*, *Microcoleus*, and *Geodermatophilus* were among the seven genera more abundant in early successional biocrusts [23]. Ecological functions, like photosynthesis, interactions with vascular plants, soil stabilization, and nitrogen fixation by biocrusts, also change with biocrust successional stage [24–27]. For example, the abundance of photoautotrophic organisms may decline over time [28].

Burned biocrust may also have an altered microbial community composition. Recent research in a cold desert of the southwestern United States found that 1 year after a fire burned biocrusts had fewer cyanobacteria and were dominated by chemoheterotrophic bacteria and fixed less nitrogen [29]. However, there is little understanding of how biocrust microbial communities respond to fire in grasslands, despite fire being an important ecological disturbance in this ecosystem [30]. Previous work has shown that some biocrusts and their functions can survive low-severity fires, which are common in grasslands [31–33]. Despite this, fire may ultimately reduce the total biocrust cover depending on the biocrust type and the time since a fire [21]. However, there is little research on the impact of fire on the biocrust microbial community which may differ from previous work emphasizing biocrust cover and macroscopic community composition.

Here, we studied the effect of prescribed fire and wildfire on biocrust community composition and ecological function within a California coastal grassland. We had two objectives:

1. To determine the effect of fire on biocrust microbial community composition.
2. To determine the effect of fire on the biocrust functional gene profile and measured ecological function.

We analyzed the microbial community composition and functions between prescribed fire plots measured 1 year after a fire and wildfire plots measured 6 years after a fire and compared them to unburned control plots. We predicted that there would be a difference in the biocrust microbial community composition between the burned communities and their respective controls. Since the prescribed fire and wildfire occurred in different years, we predict that the wildfire community composition and function will be more similar to the controls because it has had a longer time to recover. Six years may be an adequate timeframe to observe microbial recovery after a wildfire [13]. Both the wildfire and prescribed fire biocrusts are expected to have fewer gene copies relating to ecosystem functions such as chlorophyll biosynthesis, nitrogen fixation, and exopolysaccharide biosynthesis, suggesting reduced ecosystem function. The changes in ecological function will be quantified using physiological measurements. This study improves our understanding of how microbial biocrust communities within California coastal grasslands respond to fire and provides insights on management strategies to improve fire recovery of grassland ecosystems.

## Methods

**Fig. 1:**
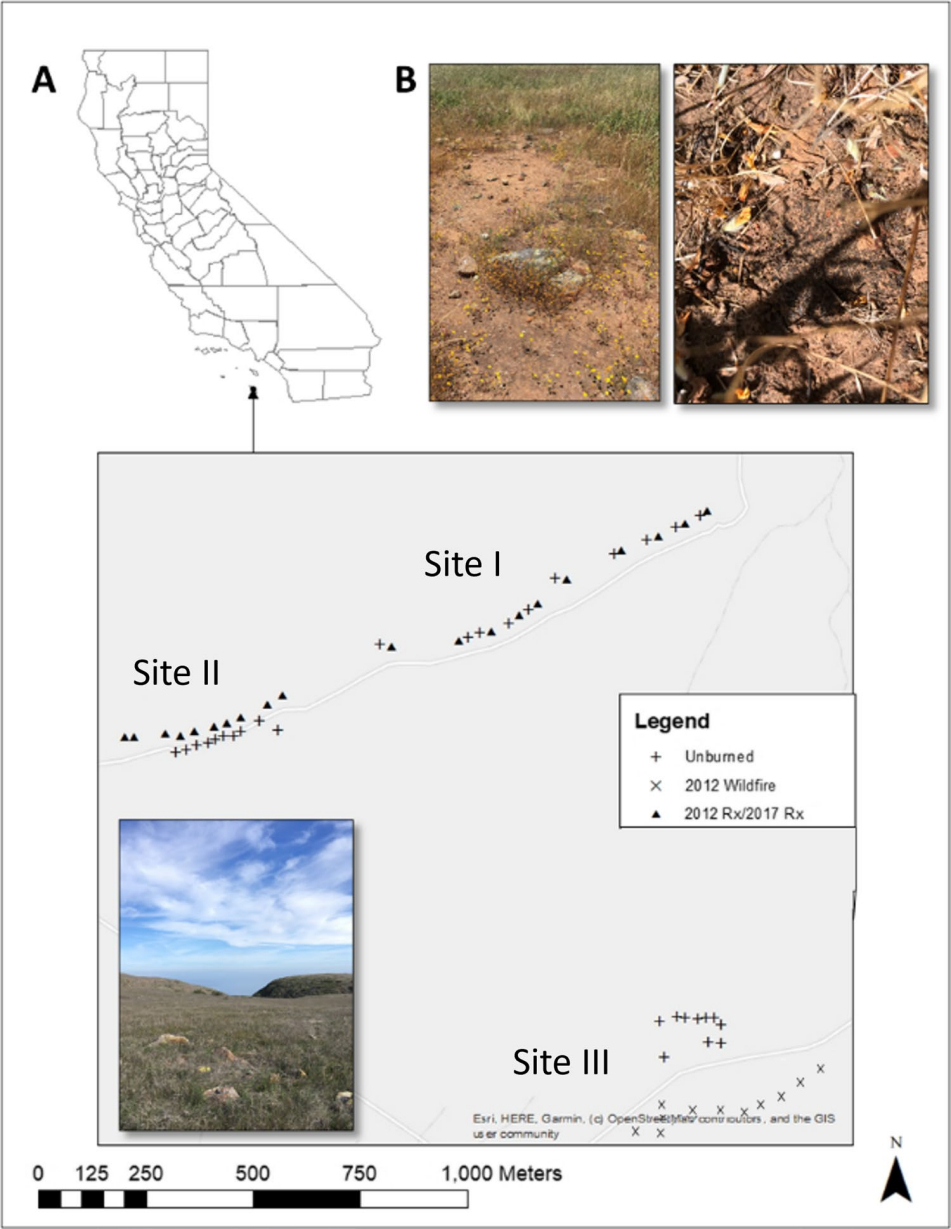
**A** Map of the plots on San Clemente Island with a photo of site I. Ten plots each 10m^2^ were burned in a prescribed fire in sites I and II in 2012 and 2017. Adjacent unburned plots were established as controls. In site III, we collected biocrust from ten locations within the 2012 wildfire perimeter and included ten unburned plots outside of the wildfire perimeter. **B** Typical habitat for SCI biocrusts. Biocrusts on SCI are often found growing in large patches in open spaces or nestled between dense plant clumps

The study area (32°53′58.76″N, 118°29′24.46″W) was located on San Clemente Island (SCI), the southern-most island of the Channel Island chain off the coast of southern California (Fig. 1). Biocrusts are an important component of the Channel Island ecosystem [34]. The vegetation community is a mix of perennial and annual grasslands [35]. The climate is Mediterranean Dry Summer Subtropical with an average of 20.5 cm of annual rainfall from 2005 to 2016, with much of the rainfall occurring between November and April [36] and persistent coastal low clouds and fog in the warm season [37]. Natural fires are rare on the island with only 3 lightning-caused fires in 140 years and there is no evidence of historical Indigenous burning [36]. However, a wildfire did occur on SCI in May 2012 and prescribed burns have been used to facilitate the growth of native plants [35].

### Plot Design and Fire History

The grassland soils are clay derived from volcanic substrates, primarily alfisols and vertisols [35, 38]. Perennial Grasslands East (site I) and Perennial Grasslands West (site II) were burned in a prescribed fire in 2012 and 2017 [35]. The sampling occurred one year after the 2017 prescribed fire. The burns consumed most vegetation in the plots; in both years, the average area burned exceeded 95% (Keeley unpublished data). Within each site, ten plots were burned, and ten adjacent unburned plots were established. The boundaries of the 10 m^2^ plots were treated with the monoammonium phosphate and ammonium sulfate-based fire-retardant Phos Chek [39]. Ten plots were 100% burned in the Ranch Canyon (site III) wildfire in May 2012 and the sampling occurred 6 years after the fire. Ten unburned plots were established outside of the wildfire perimeter (Fig. 1) [35]. Characteristic of grassland fires with low fuel loads, the prescribed fires and wildfire were determined to be low severity [31]. Plots within sites I and II were established by the United States Geological Survey to determine the impact of prescribed fire on native vegetation in 2012 and represent the typical soil type and vegetation of SCI grasslands [35]. Wildfire plots were established in an area with similar soil and vegetation to sites I and II and plots were approximately 10 m apart, accounting for large rocks that we did not want included in the plots. The site III control plots were established on the other side of a road that did not burn in the wildfire. Despite being similar severities, the time since fire—1 and 6 years—may lead to different results between the sites.

### Biocrust Sampling

In April 2018 and May 2019, we collected approximately 20 g of biocrust from each plot by scraping the biocrust off with a sterile pie server and placing each sample into a sterile plastic bag. To avoid sampling the bulk soil, we only collected the top centimeter. We avoided collecting samples growing directly adjacent to large rocks, or under shrubs and cacti. All biocrusts were classified as cyanobacteria-dominated based on visual assessments, though lichen and bryophytes were present in low abundance. At the time of sampling, cyanobacteria-dominated biocrusts were the most common biocrust type within the plots. These biocrusts were used for all subsequent analyses.

### DNA Extraction and Shotgun Metagenomics

In April 2018, we collected biocrust samples for DNA extraction from four plots within each site and treatment resulting in 24 total samples (Table 1). The biocrust samples were stored in 15-mL polypropylene tubes on ice then moved to the − 80 °C freezer. We extracted DNA using a Qiagen DNeasy PowerSoil Pro DNA extraction kit with an added proteinase K incubation to improve DNA yield [40] with > 20 µL of DNA per sample. Libraries were created by the Genome Center at UC Davis where they were quality checked with the Bioanalyzer QC. Samples were sequenced using an Illumina MiSeq with 250 read in both directions. Initial taxonomic and functional classifications for the shotgun metagenomic data were determined using the MG-RAST pipeline Version 4.0.3 [41]. We uploaded the sequences in FASTQ format to MG-RAST and used the default settings which removes sequences with less than 75 bp, artificial replicates, human sequences, and sequences with a Phred score below 15. The average number of base pairs per sample was 83 Mbp (standard deviation 37, 170,485) with a total of 5,773,395 sequences and an average of 240,550 sequences per sample. Significant matches had a sequence identity of over 15 amino acids and an *e*-value < 10^−5^. Taxonomy was determined using the “Representative Hit” classification with the RefSeq database as a reference which is based on the first hit in the homology search and the first annotation for that hit in the database. This method is appropriate to compare taxonomic and functional profiles between metagenomes [41]. For functional classification, MG-RAST classifies sequences into subsystems which are grouped into hierarchal categories. “Functions” is the most detailed category (e.g., assimilatory nitrate reductase large subunit (EC:1.7.99.4) to “Level 1” which is the least detailed category (e.g., Nitrogen Metabolism). “Level 2” and “Level 3” are intermediate categories.

**Table 1:** Counts are numbers of paired samples within site × treatment. Sites I (Perennial Grassland East) and II (Perennial Grassland West) were prescribed burns while Site III (Ranch Canyon) was a wildfire

	Site:	I (PGE)		II (PGW)		III (RC)	
	Treatment:	Unburned	Burned	Unburned	Burned	Unburned	Burned
*Response variable:*	Metagenomics	4*	4*	4*	4*	4†	4†
	Nitrogen fixation (2018)	6*	6*	6*	6*	6^†^	6^†^
	Nitrogen fixation (2019)	6**	6**	6**	6**	6^††^	6^††^
	Chlorophyll content (2018)	10*	10*	10*	10*	10^†^	10^†^
	Chlorophyll content (2019)	10**	10**	10**	10**	10^††^	10^††^
	EPS content (2018)	10*	10*	10*	10*	10^†^	10^†^
	EPS content (2019)	10**	10**	10**	10**	10^††^	10^††^

^*^1 year since prescribed burn,**2 years since prescribed burn, ^†^6 years since wildfire, ^††^7 years since wildfire

We used applications developed for KBase [42] to assemble and annotate metagenome-assembled genomes (MAG). Reads from both the burned and control samples which were quality checked using Fast-QC [43] combined into one read library and trimmed using Trimmomatic [44] to remove the TruSeq3-PE-2 adapters with the default Trimmomatic settings. Trimmed reads were assembled using metaSPAdes with a minimum contig length of 1000 bp [45]. Assemblies were binned using MetaBAT2 [46] and the resulting genome quality was checked with CheckM [47]. Assemblies were classified using GTDB-tk [48] classify and annotated with Rasttk [49]. The quality of the MAG was assessed by assessing the completeness and contamination scores [50]. Using HMMER3 within KBase [51], we extracted the 16S rRNA genes and used them to create a bootstrapped maximum likelihood tree. We used reference sequences from NCBI and MEGA 11 to create the tree with 500 iterations [52]. The tree was annotated using FigTree [53].

### Physiological Analyses

For nitrogen fixation, chlorophyl content, and EPS content, we used biocrusts collected from each site in 2018 and 2019. For chlorophyll and EPS, we used one sample from each plot (*N* = 60 per year) and measured chlorophyll content three times for each sample. For nitrogen fixation, we used six plots for each site, treatment, and year (*N* = 36 per year) (Table 1).

Total chlorophyll concentration was determined using a modified DMSO extraction method [54]. All the samples were dried in a drying oven at 60 °C for 24 h and the dry weight was measured, the average dry weight was 3.35 g. Then, each sample was sprinkled with distilled water to activate the biocrust organisms. Each biocrust sample was added to a 15-mL tube with a spatula tip of CaCO_3_. We added 6 mL of DMSO to each tube and placed it in a hybridization incubator with Isotemp Rotisseries (Fisher Scientific) where the tubes were incubated at 65 °C for 90 min and continuously rotated. Following this, the supernatant was transferred to a separate vial, and 6 mL of DMSO was added to the original sample tubes for the second extraction cycle. Both supernatants were pooled and centrifuged for 10 min at 3000 × *g* at 15 °C. Then, 100 µL of the extractant was loaded into a 96-well plate in triplicate and the absorption was measured on a SpectraMax 190 microplate reader (Molecular Devices, San Jose, CA) at 648, 665, and 700. Chlorophyll *a* + *b* concentrations were calculated using the following equation.

1 $$Chl a+b=\left[\left({A}_{665}-{A}_{700}\right)\times 8.02+\left({A}_{648}-{A}_{700}\right)\times 20.2\right]\times \mathrm{DF}\times S$$

where *A*_*x*_ is the absorbance at wavelength “*x*”; DF is the dilution factor; *S* is the amount of solvent (ml).

Exopolysaccharide (EPS) concentration was measured using the weak acid extraction method [55]. Five grams of biocrust for each sample was air-dried and added to 100-mL Nalgene bottles containing 50 mL 0.5 M H_2_SO_4_ and autoclaved for 60 min at 121 °C and 103 kPa. Following this, each sample was centrifuged at 5200 × *g* for 20 min at 4 °C. The supernatant was transferred to 50-mL tubes which were immediately stored at − 80 °C. Total EPS content was determined by combining 400 μl of the supernatant, 10 μl of 80% phenol, and 1 mL 0.5 M sulfuric acid [56]. After 10 min, the plates were read on the microplate reader at 490 nm using 10 mg/100 mL and 1 mg/100 mL glucose as standards.

To determine nitrogen fixation, we placed 0.5 g of biocrust samples inside desiccators, each sample was approximately 1 cm thick. All samples were hydrated with 3 mL distilled water 24 h before incubation. This was enough water to fully saturate the biocrust, without the water pooling. The desiccators were vacuum sealed and ^15^ N was injected using a gas bag attached to a syringe. We added enough labeled ^15^ N to create an 8.3% ^15^ N atmosphere. The desiccators were placed in a growth chamber at 25 °C with a radiance of 20.3 μmol m^−2^ s^−1^, and with 12 h of light for 2 weeks [57]. Samples were then removed from the desiccator and ground up for analysis on the mass spectrometer. For each sample, we also analyzed the baseline ^15^ N from samples that were not exposed to the enriched atmosphere from the same biocrust sample (*N* = 72). Nitrogen fixation was calculated using the following equation:

2 $$Y=\frac{\mathrm{atom}\%{}^{15}N\;\mathrm{exess}}{100}\times\frac{\mathrm{Total}\;\mathrm N\times10^9}{t\times28}\times\frac{100}{\%{}_{}{}^{15}\mathrm N\mathrm{air}}$$

where Y (nmol N × mgDW^−1^ day^−1^) is the rate of N_2_ uptake, atom% ^15^ N excess is the difference between the atom% ^15^ N of the incubated samples and the control samples, total N is the total nitrogen in the incubated samples (g × 100 gDw^−1^), *t* is the incubation time (hours), and 28 is the relative molecular mass of N_2_(g mol^−1^), and %^15^ N air is the percentage of ^15^ N out of the total amount of N gas in the desiccator (8.3%). Atom% for both incubated and control were calculated using Eq. 3. δ15N is the stable isotope ratio of ^15^ N: ^14^ N and 0.3363 is the standard for the percent of ^15^ N of all the nitrogen gas in the atmosphere.

3 $$\mathrm{atom}\%{}{}^{15}N=\left(\frac{\delta {}{}^{15}N}{1000}+1\right)\times 0.3663$$

### Statistical Analyses

All analyses were performed in R version 4.02. Separate generalized mixed linear models were used to look at the results of the physiological analyses using the lme4 package [58]. For all the models, we examined the fire type (prescribed, wildfire) and their respective controls. In the linear models, the samples were considered to be paired (burned and unburned) and the pairs were included as a random effect and significance was assessed using Tukey post hoc tests using the “emmeans” package [59]. We calculated the Shannon-diversity index and richness of the genus level for all the biocrust metagenomes using the package “vegan” [60]. We compared the differences in these measurements between treatment and site using the “Anova” function in the “car” package then used a Tukey post-hoc test [61]. Additionally, we calculated the Bray–Curtis Dissimilarity distances based on the genus for each community and performed a PERMANOVA using the “adonis2” function within “vegan” to determine the similarities between community make-up [60]. These data were visualized using non-metric multidimensional scaling (NMDS) plots. Differences between gene abundance of specific taxa and functional genes were determined using the “Anova” function and Tukey post hoc tests [62]. We used “phyloseq” and “igraph” to create a network of the microbial community [63, 64]. We performed this analysis for the burned and unburned communities separately. All codes can be accessed via GitHub at https://github.com/briannepalmer/San-Clemente-Biocrust-.

## Results

**Fig. 2:**
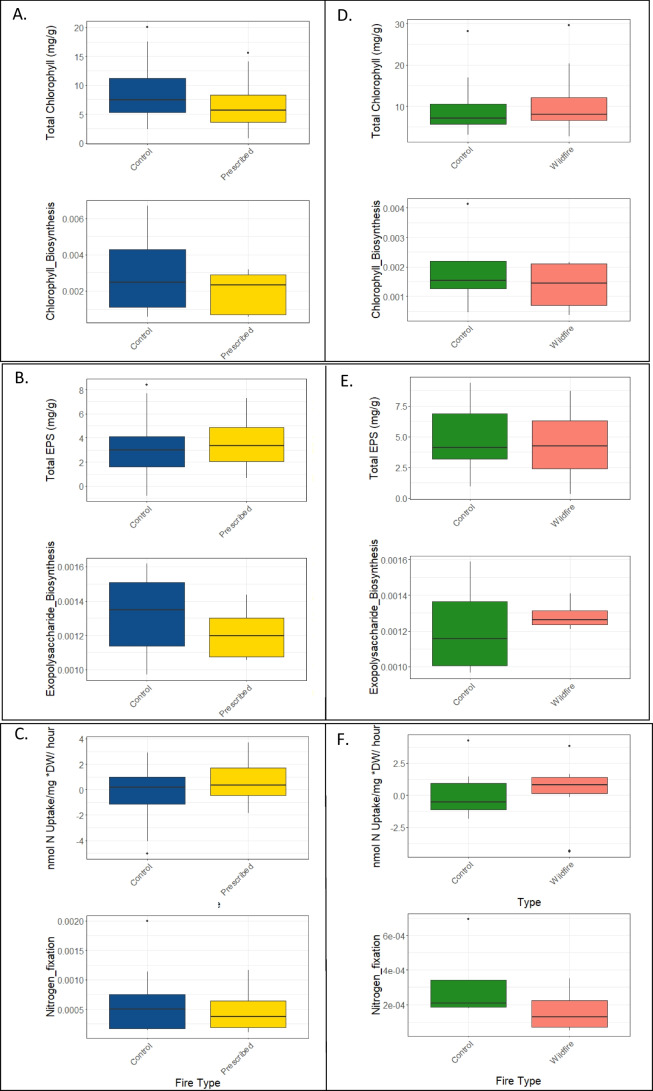
**A** The total measured chlorophyll for each fire type and their respective controls; **B** the relative abundance of nitrogen fixing genes; **C** the measured EPS content; **D** the relative abundance of the exopolysaccharide biosynthesis genes; **E** the measured nitrogen fixation; **F** the relative abundance of nitrogen fixing genes

The average chlorophyll content, EPS content, and nitrogen fixation rates for the biocrusts were 6.89 (± 5.93) mg·g^−1^, 3.79 (± 2.24) mg·g^−1^, and 0.15 (± 1.85) nmolN·mg^−1^·g·h^−1^. There was more chlorophyll in the controls compared to the prescribed burn plots in sites I and II (Table 2, Fig. 2), though there was no difference in chlorophyll content between the wildfire plots and the adjacent controls in site III. The total EPS concentration (mg/g) and the nitrogen fixation rates did not differ between the fire types and their corresponding controls (Table 2, Fig. 2).

**Table 2:** Results of the Tukey post hoc tests for all the functions measured in the lab. Chlorophyll measurements include both chlorophyll a and b. Bold values indicate a *p*-value < 0.05. Sites I + II were burned in a prescribed fire and site III was burned in a wildfire

Variable	Regression coefficient	Std. error	*P*-value
Chlorophyll			
Sites I + II–control	− 2.42	1.001	**0.016**
Site III–control	1.75	1.93	0.36
Exopolysaccharides			
Sites I + II–control	0.341	0.484	0.894
Site III–control	− 0.383	0.684	0.943
Nitrogen fixation			
Sites I + II–control	− 0.058	0.424	0.999
Site III–control	− 0.540	0.640	0.830

### Microbial Community Composition

**Fig. 3:**
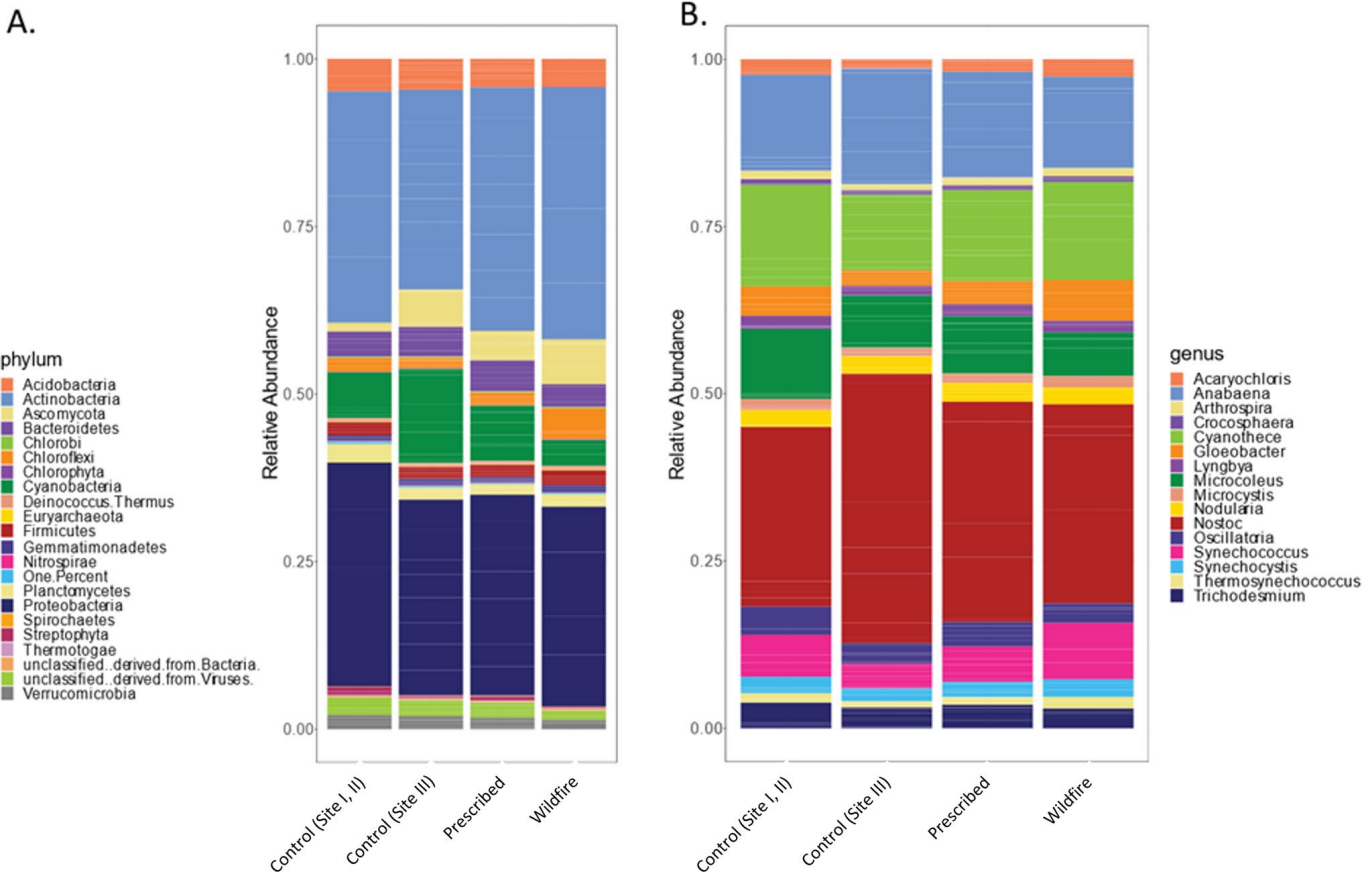
**A** Relative abundance of each phylum. Phyla that had less than 1% relative abundance are grouped. **B** Relative abundance of cyanobacteria genera

The total metagenomic dataset consists of 0.5% Archaea, 2.1% Viruses, 6.3% Eukarya, and 91.1% Bacteria. There was no difference in the relative abundance between the controls, prescribed fire, and wildfire treatments for Archaea, Bacteria, or Eukarya and the control plots had a greater mean proportion of viruses than the wildfire plots (*P* < 0.0001). The most abundant phylum across all samples were Actinobacteria (32.3%), Proteobacteria (30.1%), and Cyanobacteria (8.7%) (Fig. 3). *Nostoc* was the most abundant cyanobacteria genus, and the abundance of all cyanobacterial genera did not vary between treatment and site, nor were there significant differences in the microbial community composition between sites.

**Fig. 4:**
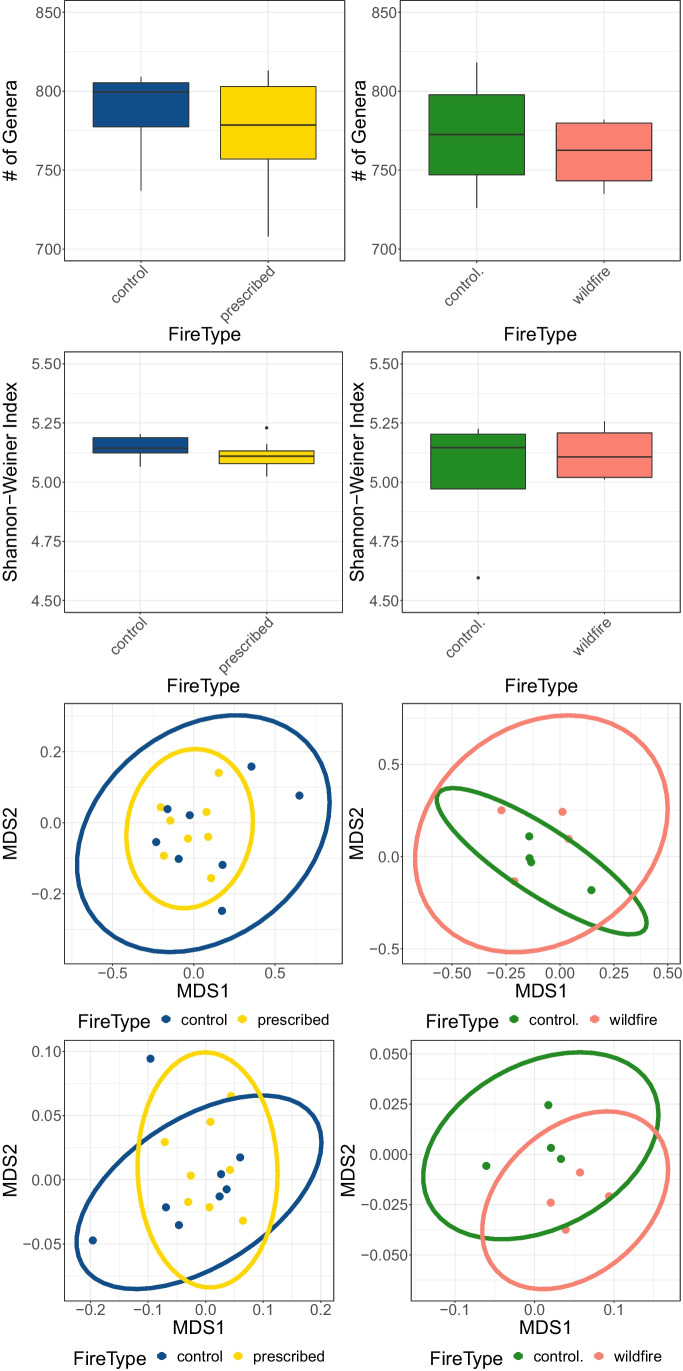
**A** Richness values for each treatment. There was no difference in the total number of genera within each treatment. **B** Shannon diversity index for each treatment. There was no difference in the Shannon diversity between treatments. **C** NMDS plot based on genera in each treatment. The community composition between the treatments was similar. **D** NMDS plot based on the functions level of the SEED database. The functional genes present are similar between the wildfire and prescribed fire and the prescribed fire and controls. There is a difference in the functional genes present between the wildfire and control samples (*P* = 0.043)

Based on the PERMANOVA and subsequent post hoc analyses, there was no difference in the microbial community composition between the wildfire, prescribed fire, and the control plots (Table 3, Fig. 4). Diversity and richness were also similar across treatments (Fig. 4). However, seven genera varied in their relative abundance between the prescribed fire and the control, and nine genera varied between the wildfire and the control (Figure S2). The wildfire and control samples also had a different composition of functions based on the Subsystem annotations (*P* = 0.025, *R*^2^ = 0.223, Fig. 4, Table 3). There was no difference in the number of genes for nitrogen fixation, exopolysaccharide biosynthesis, or chlorophyll biosynthesis between treatments as indicated by the level 2 and level 3 functional gene characterizations from the Subsystems database in MG-RAST. However, twelve function sequence copies were more abundant in the prescribed fire samples compared to the control and seven were more abundance in the wildfire compared to the control (Fig. 6).

**Table 3:** Results of the PERMANOVA for the taxonomic community on the genus and function levels. Bold values represent p-values < 0.05. Sites I + II were burned in a prescribed fire and site III was burned in a wildfire

Pairs	Df	SS	*F* model	R2	*P* value
Genus level					
Sites I + II–control	1	0.0280	0.935	0.0626	0.415
Site III–control	1	0.0296	1.19	0.165	0.313
Function Level					
Sites I + II–control	1	0.00597	0.930	0.623	0.441
Site III–control	1	0.0061	1.72	0.223	**0.025**

### Network Analysis

**Fig. 5:**
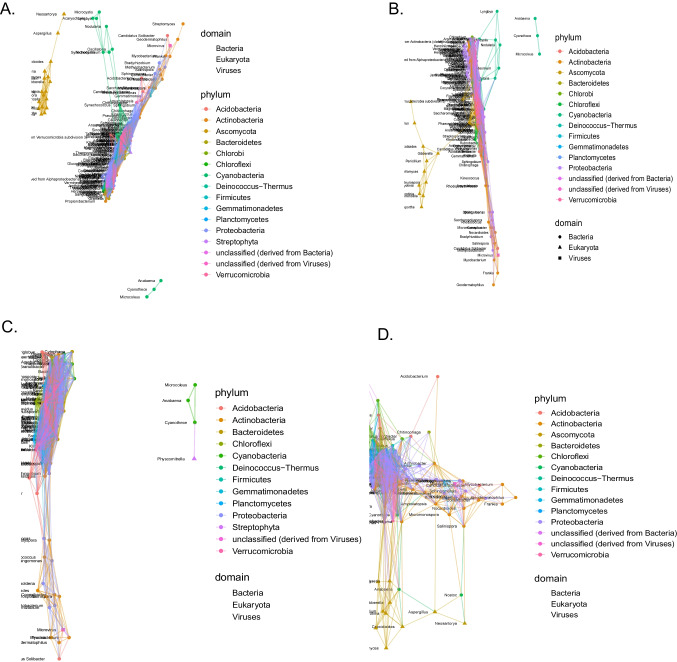
**A** Networks for control samples from sites I and II (*n* = 8) (**B**), prescribed burn samples from sites I and II (*n* = 8) (**C**), control samples in site III (*n* = 4) (**C**), and wildfire samples from site III (**D**). Each point represents a genus, the shapes depict different domains, and the colors represent different phyla

Each treatment (prescribed, wildfire, and the two controls) have different network shapes (Fig. 5). The prescribed fire control had the most network connections (154) and the wildfire control had the least (92) (Table S1). Across all networks, Proteobacteria and Actinobacteria had the most connections (Table S1).

Both the prescribed burn, and the associated controls, have three genera of cyanobacteria that are separate from the network—*Anabaena*, *Cyanthece*, and *Microcoleus*. The eukaryotic Ascomycota formed a separate network in the control plot (Fig. 5A). In the prescribed burn community, the same three cyanobacteria genera formed a separate network. However, Ascomycota, though still primarily connected to each other, have a connection with the larger network (Fig. 5B). The wildfire controls also had the same three cyanobacteria genera forming their own network with an addition of *Physcomurella*, in the phylum Steptophyta. This network lacks the large cyanobacterial network shown in the prescribed fire and other control networks (Fig. 5C). The wildfire network does not have the network of three isolated cyanobacteria; rather, it is all connected. It still has a group of Ascomycota which are connected to each other, but this is also connected to the larger network (Fig. 5D). The cyanobacteria genera *Anabaena* and *Nostoc* are connected to the Ascomycota (Table S1). The prescribed fire network had more connections to Actinobacteria; however, the associated controls had more connections to Ascomycota, Bacteroidetes, Cyanobacteria, and Proteobacteria. The wildfire network also had more connections to Actinobacteria compared to the associated controls and more connections between Ascomycota. The controls associated with the wildfire did not have any connections between Ascomycota but did have more connections to Bacteroidetes, Cyanobacteria, Proteobacteria, and Verrucomicrobia (Table S1).

### Metagenome Assembled Genome

The MAG was created with all 24 metagenomes because no MAGs were found when we assembled based on treatment. We found one medium quality MAG that is 77.78% complete with 3.14% contamination within the family Pseudonocardiaceae. The GC content is 66.34%. The most abundant functions in the Rasttk annotations were related to amino acids and carbohydrate processing, and other functional categories that are necessary for cell function. Interestingly, there were 7 genes for oxidative stress, 10 genes for heat shock, 28 for capsular and extracellular polysaccharides, 10 genes for ammonia assimilation, and 7 for nitrate and nitrite ammonification. Using the 16S ribosomal RNA genes from within the MAG and reference sequences from NCBI and the bootstrapped maximum likelihood tree, we placed the MAG in a clade with *Microbispora bispora* (Figure S1).

## Discussion

This is the first study to analyze biocrust metagenomes from a coastal grassland. It provides insight into how biocrusts recover from fire which can inform restoration and land management in the future—particularly due to the outsize role biocrusts play in ecosystem function [15–17, 65].

Our results provide insight into the microbial community composition and function of biocrusts 1 year after a prescribed fire, 6 years after a wildfire, and from plots that did not experience fire. Interestingly, the community composition and measured functions were similar between both fire types and their respective controls. However, there were some differences in the relative abundance of function sequences between the fires and their controls. Additionally, there were differences in the microbial networks. This has implications for understanding how grassland biocrust communities respond to fire.

After 1 year, the prescribed fire microbial community is similar to the unburned controls, suggesting a level of recovery one year after the fire. Furthermore, 7 years after the wildfire, the microbial community is similar to the control community, as expected, but there are differences in the relative abundance of functional genes that suggests there may be legacy effects of the fire impacting microbial function. This should be explored further.

### Measured Functions Were Similar Between Treatments

The chlorophyll content may be used as a proxy for the relative abundance of photosynthetic organisms [54]. In sites I and II, the control samples had greater chlorophyll content than the prescribed burned samples. Previous studies had similar findings. For example, after a grassland fire in New Mexico and the Palouse prairies, burning reduced chlorophyll *a* content in biocrusts up to 1 year after the fire [32, 66], although in the case of the wildfire, 7 years may be sufficient time for the chlorophyll to reach the same level as the unburned biocrust. Interestingly, previous literature suggests that cyanobacteria are less resistant to fire based on pigment analyses like chlorophyll *a* [31, 32] compared to this study. In this study, we found similar amounts of chlorophyll *a* between treatments, all the biocrusts were classified as cyanobacteria-dominant, and there was no difference in the relative abundance of cyanobacteria between the treatments (Fig. 2). However, chlorophyll measurements provide an estimate of how much of the sample is photosynthetic; it is not a direct measure of photosynthetic activity. Future work should directly measure the effect of fire on the photosynthetic capacity of biocrusts.

EPS is often used as a measure of soil stability [67]. Since EPS concentrations were similar between the treatments in this study, this suggests that the ability of the biocrusts to hold the soil aggregates together is the same in all the treatments. This is important in terms of fire ecology and management because increased erosion is a significant effect of wildland fires [68]. All plots—prescribed, wildfire, and both controls—were dominated by cyanobacteria biocrust (Palmer et al. in prep) and similar biocrust types have similar EPS content [69]. Therefore, it is not surprising that EPS was similar between two similar biocrust types, but what is important in terms of restoration is that the EPS content within the biocrusts in the prescribed fire plots recovered to unburned levels within a year.

Like EPS, nitrogen fixation often increases with biocrust age [69]. Since the crusts in both fire treatments and the controls were all classified as cyanobacteria-dominated, it is unsurprising that there is no difference in the nitrogen fixation rates between treatments. A previous study in the New Mexico shortgrass-steppe found that nitrogen fixation was initially reduced 1 year after a fire, but was similar to the unburned sample after 2.5 years [66]. This is similar to our results, although we found similar nitrogen fixation rates 1 one year after the prescribed burn. Our calculated nitrogen fixation rates are similar to those documented previously in biocrust [65].

### Microbial Community Composition Was Similar Between Both Fires and Their Controls

The abundance of each phylum on SCI is similar to estimates from previous biocrust sequencing studies and contains several of the bacterial genera previously detected within biocrusts including *Acaryochloris*, *Anabaena*, *Arthospira*, *Conexibater*, *Cyanothece*, *Geodermatophilus*, *Leptolyngbya*, *Lyngbya*, *Microcoleus*, *Micromonospora*, *Mycobacterium*, *Nitrospira*, *Nocardioides*, *Nostoc*, *Pyyamimonas*, *Rubrobacter*, *Sphingomonas*, *Streptomycetes*, *and Synechoccus* [23, 70–73] (Table S2)*.*

Contrary to our hypotheses, there was no difference in the microbial community composition between burned and control plots at any of the phylogenetic levels. We were surprised by the composition of the cyanobacterial communities, as these communities are studied in a variety of dryland ecosystems. We expected *Microcoleus*, traditionally an early successional filamentous bacterium [74], to be the dominant genus in the burned plots, but found the community dominated by nitrogen-fixers like *Nostoc*, *Anabaena*, and *Cyanothece*. The abiotic environment, such as soil temperature and pH, can determine what cyanobacteria genera are dominant [75]. In biocrusts in Brazil, nitrogen-fixing cyanobacteria were more abundant in arid environments (characterized as 200–800 mm of rainfall) while filamentous cyanobacteria were more abundant areas with greater rainfall [75]. SCI receives between 200 and 500 mm of rainfall annually [36] and has persistent coastal fog in the warm season [37]. The combination of low rainfall and using fog as a water source when it is available [76, 77] may have impacted the composition of cyanobacteria in the SCI biocrusts.

We compared our cyanobacterial sequences to previous cyanobacteria inventory on the nearby San Nicolas Island (SNI). Unlike our study, the SNI inventory used culture-dependent methods to identify community members [34]. The only genera that are common between this study and the inventory of SNI were *Microcoleus*, *Nostoc*, and *Synechocystis*. This may be due to the difference between culture-independent and dependent techniques. Additionally, several of the genera identified in the metagenomes are known from aquatic rather than terrestrial environments. Other reasons for this finding may be the maritime climate and proximity to the ocean or due to an underrepresentation of terrestrial cyanobacterial genomes in the SEED database [73].

One explanation for the similarities between the burned and control communities is that the bare soil after the fire could be quickly recolonized by a nearby source population. Due to the setup of our plots, with burned plots adjacent to unburned areas, there was a nearby source population that could have recolonized after the fire. Cyanobacteria, the initial colonizers, readily aerosolize and can travel long distances and land on the soil to form a biocrust [78]. Another explanation may be that the biocrust microbes were able to withstand the fire. Deeper soil layers (~ 2 cm deep) are protected from the heat of fire [79] and biocrust microbes have a demonstrated ability to rehydrate quickly after severe drought stress which may be valuable after fire [80]. Additionally, the EPS produced by cyanobacteria and other biocrust microbes can allow the microbes to survive extreme temperatures [81] and terrestrial cyanobacteria can survive temperatures of 100 °C [82] which is within the range of soil temperatures during a low severity wildfire 1 cm below the soil surfaces [14].

Previous work on biocrust recovery has highlighted that it may take years, decades, or even centuries for biocrusts to recover from fire [18]. However, more recently, a new understanding of resistance, resilience, and recovery of biocrust microbial communities after physical and climate disturbances is emerging [83]. The speed of biocrust microbial recovery after fire our results demonstrate could be a phenomenon unique to the Mediterranean climate. Fog and low-cloud formation on the SCI are important in structuring plant communities [37] and may be important in the recovery of biocrust communities as well, due to the moisture in the clouds that could aid in the recovery of desiccation-tolerant biocrust organisms.

### Difference in Functional Gene Sequences

Functional gene composition did differ between the wildfire and the control samples. Changes in the functional gene profile without changes to the taxonomical composition suggest that there may be unaccounted for diversity below the taxonomic levels addressed in this study—at the species or strain level. Shotgun metagenomics often does not provide accurate information at lower taxonomic ranks [73].

**Fig. 6:**
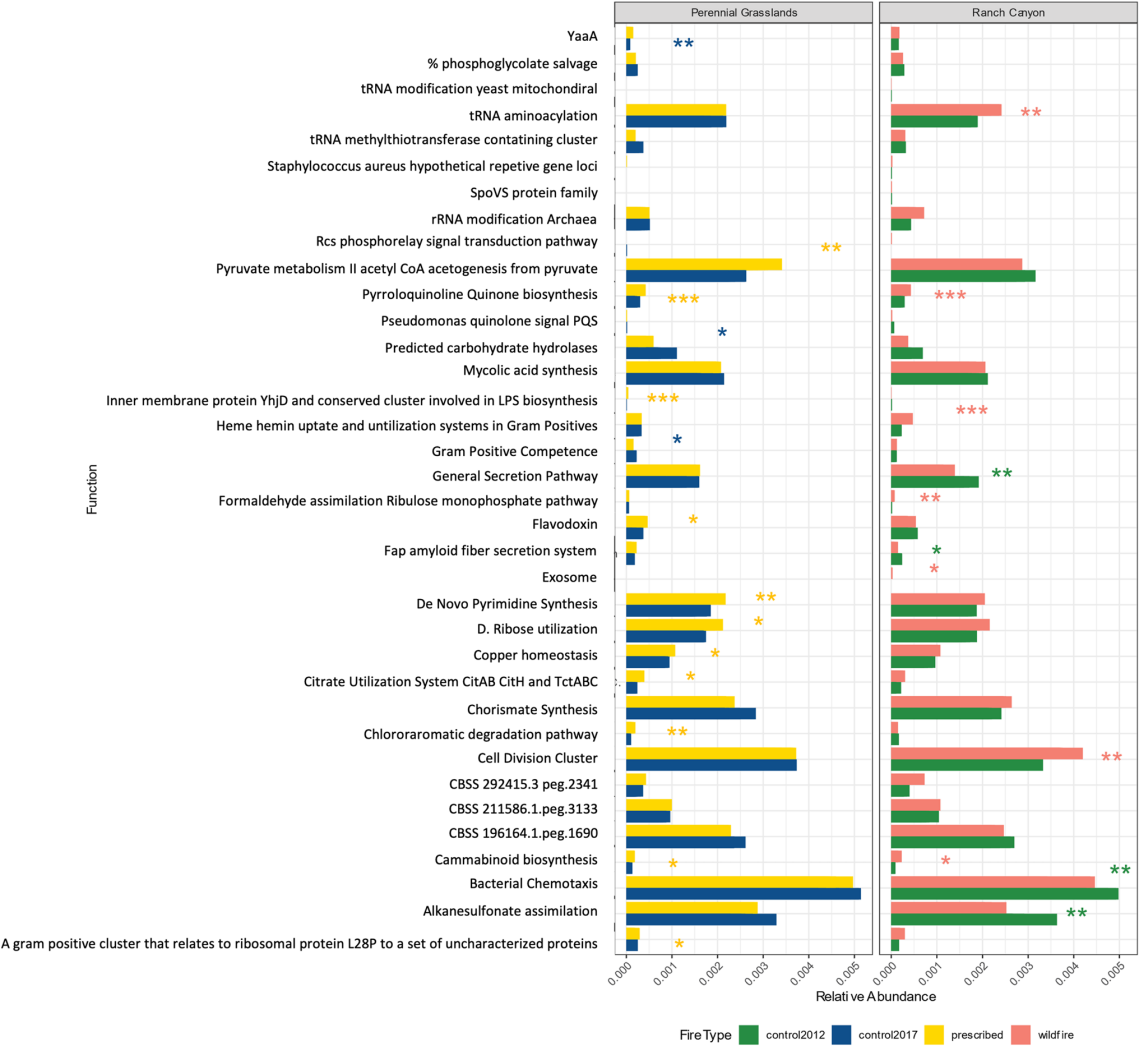
The functional gene sequences that varied between the treatments. The color of the * indicates which treatment had a greater relative abundance. Perennial grasslands include the prescribed fires in sites I and II and site III includes the wildfire. The * represents a *p*-value < 0.05, ** represents a *p*-value < 0.01, and *** represents a *p*-value < 0.001

Although it was not a significant difference in the functional gene composition based on the PERMANOVA, 16 functional gene sequences varied between the prescribed fire and the controls and 12 of the sequences were more abundant in the prescribed burn samples including cannabinoid biosynthesis and pyrroloquinoline quinone biosynthesis which were also more abundant in the wildfire samples (Fig. 6). Pyrroloquinoline quinone is a bacterial cofactor involved in redox reactions [84]. Exosome sequences were only found in the wildfire samples (Fig. 6). Exosomes are important for intracellular transport and communication [85]. The wildfire samples also had more sequences for cell division and bacterial chemotaxis suggesting that the microbes in that environment may be dividing more and have more genes to sense chemicals in the environment.

Although there were some differences in the functional gene sequences between the fire types and the controls, the key metabolic pathways and biogeochemical pathways important for the biocrust ecosystem functions were similar between the treatments.

### Insights from the Network Analysis

The network analysis revealed some differences between the treatments. Although each phylum was connected to a similar number of genera (Table S1), there are differences in the shape of the networks. For one, the Ascomycota are connected to the larger network in the prescribed fire but not the control, suggesting that although the microbial community is similar between the treatments, the microbes differ in the communities that they cooccur with. These two networks also have a group of cyanobacteria that are disconnected—*Anabaena*, *Cyanothece*, and *Microcoleus—*these are common cyanobacteria in biocrusts and often early successional [74] and previous network analyses in the Namib Desert showed that Cyanobacteria are often network hubs, not separated from the network [86]. Interestingly, in this study, the cyanobacteria and Ascomycota are not network hubs in the controls or the burned metagenomes. This suggests that in this environment, the heterotrophic microbial communities are not structured around Cyanobacteria and Ascomycota, despite their foundational role in biocrusts globally [15]. Perhaps this is due to the Mediterranean climate and the predominance of coastal fog, which may select for biocrust communities with a different community structure, though more comparisons with biocrust communities from other regions are needed. Other studies that performed biocrust network analyses found that, similar to this study, Proteobacteria and Actinobacteria can be network hubs [87] and that the prokaryotic community within biocrust becomes more similar as the biocrusts become more developed [88]. The similarities between the networks in this study provide further evidence that despite the disruption of the fires, the burned biocrusts have a similar level of development as the unburned biocrusts.

### Metagenome Assembled Genome Provides Insight into Potential Ecological Function

The MAG was placed within Actinobacteria, the most abundant phylum in the samples. Since it was created with all 24 metagenomes, we cannot draw conclusions about whether or not this MAG can survive fire or may be an early colonizer after a fire—rather, we can extrapolate that it is a common microbe in SCI biocrusts. It has gene sequences that hint at its functional role in the biocrust including genes for nitrogen cycling such as ammonia assimilation and nitrate and nitrite ammonification, although it is not a nitrogen fixer. Importantly, it has genes for EPS formation which suggest that it may play a role in soil stability and the EPS can act as a protective layer for the cell [89]. The MAG also included gene sequences for oxidative and heat stress, which would be beneficial for a microbe living on an island in a Mediterranean climate. Based on the extracted 16S sequences, the MAG is phylogenetically related to *Micobispora* (Figure S1)*.* Several species within the genera *Micobispora* are thermotolerant [90, 91]. Of course, the recovery of one MAG is interesting as this is the first metagenomic study in this ecosystem, but future studies should increase the sequencing depth to potentially find more MAGs and to be able to identify the MAG with confidence beyond the phylum level.

## Relevance to Restoration

Here we show that within 1 year of a prescribed fire and 6 years after a wildfire, the biocrust microbial community has a similar composition to unburned biocrust in a coastal grassland with cyanobacterial dominated biocrust. However, there are some differences in the gene sequences coding for microbial functions between the wildfire and control samples, even 6 years after the fire. Furthermore, each of the treatments (prescribed, wildfire, and the two controls) had different microbial networks.

This may be considered a form of passive restoration—the recovery of a community without intervention [92]. Restoring biocrusts leads to the recovery of other ecosystem functions [93]. Understanding how biocrust recovery occurs without intervention is important to understand so that time and resources may be delegated to communities with the most need for intervention. Passive restoration of biocrusts damaged by grazing occurred in the Great Basin [94], though the success of passive restoration is dependent on climate and the disturbance type [18]. On SCI, perhaps the right biotic and abiotic conditions were available to facilitate passive restoration of the biocrust community.

Furthermore, previous researchers have used biocrust microbial inoculum as a tool to aid in post-fire restoration [95, 96]. In this experiment, the biocrust community recovered from fire on its own within a year after the prescribed fire, but active restoration strategies such as cyanobacterial inoculum can quickly improve ecosystem functioning after a fire [95, 96].

This study also adds to our understanding about how biocrusts may respond to a common restoration method—prescribed fires. In this coastal grassland, prescribed fires are used to remove unwanted vegetation [35]. Biocrusts can persist after these low severity fires while maintaining key ecosystem functions. Thus, due to their role in the ecosystem, the survival of biocrusts after a fire may aid the restoration of vegetation communities. This was not tested in the present study and should be an area of future research.

Biocrusts in coastal grasslands are understudied and there is still much to learn about their macroscopic and microscopic components. Although in the face of global change and increasing fire frequency and severity globally, it is valuable to know how these diverse and ecologically important communities survive. As evidenced by this study, at least some biocrust microbial communities may reach similar microbial community composition and function after a fire. Understanding how microbial communities recover from disturbance will improve restoration of other ecosystem components. Future work should continue to address how biocrust microbial communities recover from a variety of ecological threats and seek to understand biocrust resilience in a variety of biocrust habitats.

## Supplementary Information

Below is the link to the electronic supplementary material.

Supplementary file1 Bootstrapped Maximum likelihood tree with the extracted 16S rRNA genes from the MAG and reference sequences from NCBI. The numbers are the bootstrap value. The higher the number the more confidence there is in the branch placement. Bootstrap values > 0.70 are considered well supported. (PNG 55 KB)

Supplementary file2 The genera that varied between the treatments. The color of the * indicates which treatment had a greater relative abundance. Perennial grasslands include the prescribed fires in Sites I and II and Site III includes the wildfire. The * represents a *p*-value < 0.05, ** represents a *p*-value < 0.01, and *** represents a *p*-value < 0.001. (PNG 312 KB)

Supplementary file3 The total number of connections to individual genera that each phylum has based on the treatment. (DOCX 15 KB)

Supplementary file4 A description of the taxa found in other biocrust microbial sequencing studies compared to the taxa found in the present study. (CSV 58 KB)
