# Supplementary figures and images for: Years After a Fire, Biocrust Microbial Communities are Similar to Unburned Communities in a Coastal Grassland
Source: Microb Ecol. 2022 Nov 8;85(3):1028–44. doi: 10.1007/s00248-022-02137-y (PMC10156770; doi:10.1007/s00248-022-02137-y)

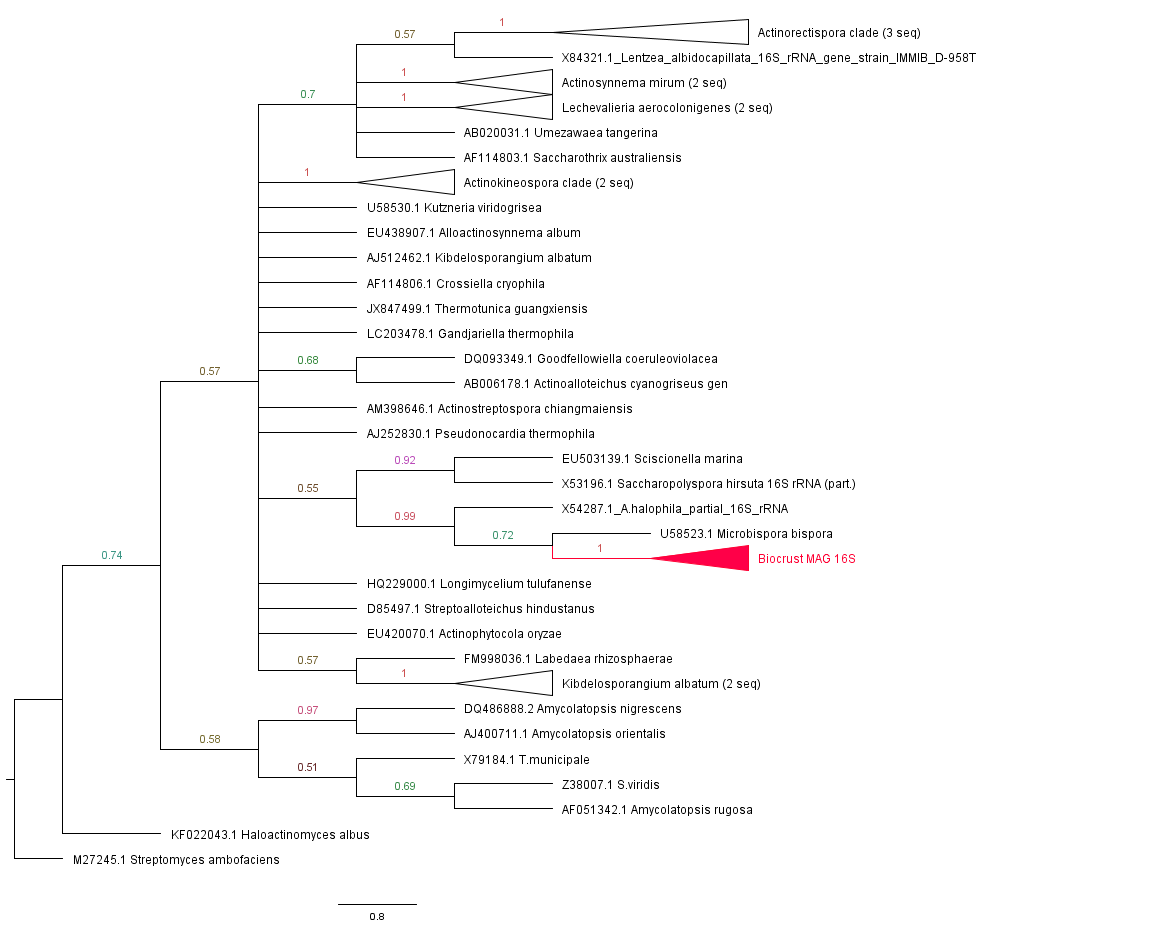

Supplement: Supplementary file 1 — Supplementary file1 Bootstrapped Maximum likelihood tree with the extracted 16S rRNA genes from the MAG and reference sequences from NCBI. The numbers are the bootstrap value. The higher the number the more confidence there is in the branch placement. Bootstrap values > 0.70 are considered well supported. (PNG 55 KB) [file 248_2022_2137_MOESM1_ESM.png]

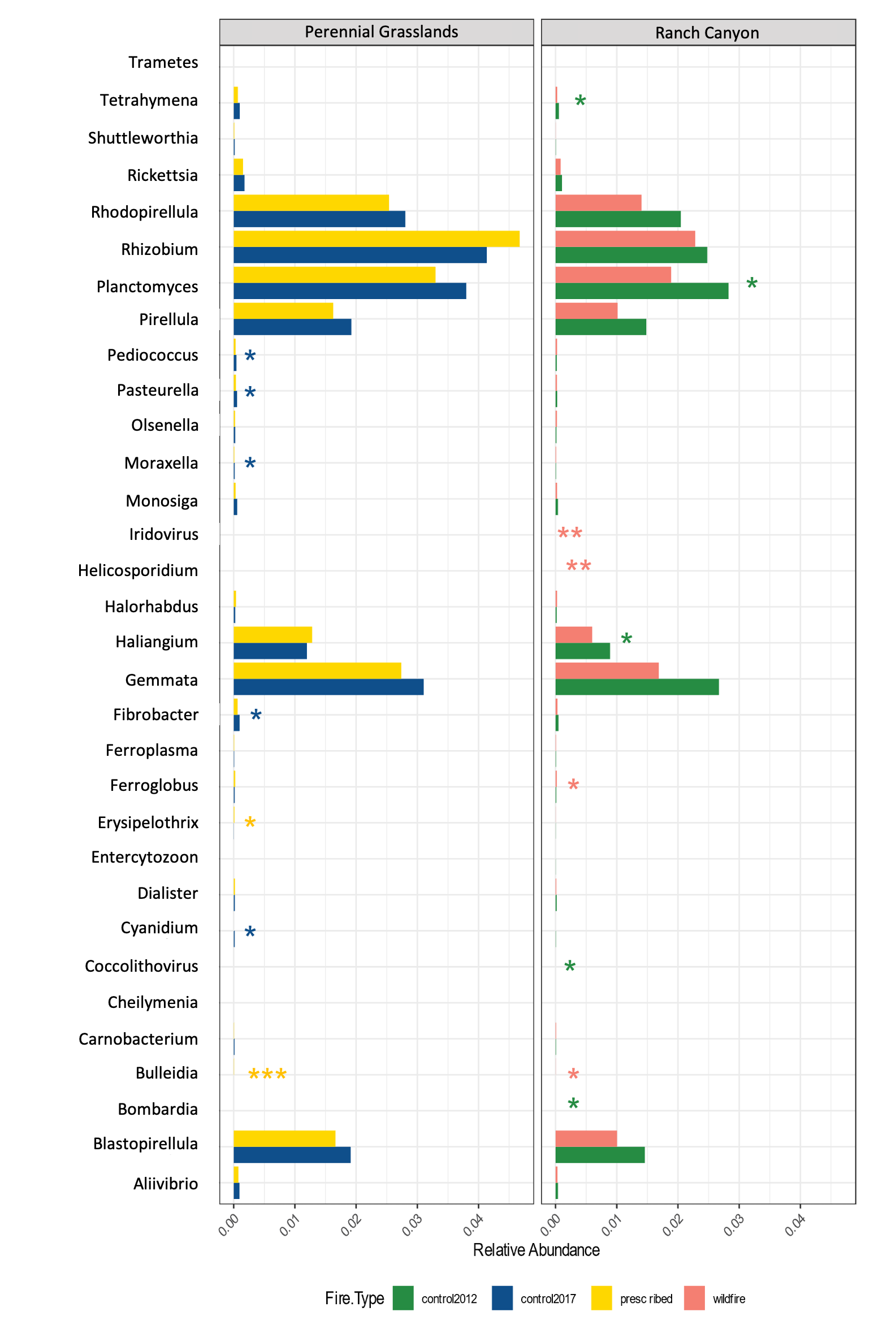

Supplement: Supplementary file 2 — Supplementary file2 The genera that varied between the treatments. The color of the * indicates which treatment had a greater relative abundance. Perennial grasslands include the prescribed fires in Sites I and II and Site III includes the wildfire. The * represents a p-value < 0.05, ** represents a p-value < 0.01, and *** represents a p-value < 0.001. (PNG 312 KB) [file 248_2022_2137_MOESM2_ESM.png]
