# Supplementary material for: Years After a Fire, Biocrust Microbial Communities are Similar to Unburned Communities in a Coastal Grassland
Source: Microb Ecol. 2022 Nov 8;85(3):1028–44. doi: 10.1007/s00248-022-02137-y (PMC10156770; doi:10.1007/s00248-022-02137-y)
Supplement: Supplementary file 3 — Supplementary file3 The total number of connections to individual genera that each phylum has based on the treatment. (DOCX 15 KB) [file 248_2022_2137_MOESM3_ESM.docx]

|  | # Genera Connected to | | | |
| --- | --- | --- | --- | --- |
| Phylum | Prescribed Fire Control | Prescribed Fire | Wildfire Control | Wildfire |
| Acidobacteria | 4 | 4 | 4 | 4 |
| Actinobacteria | 36 | 37 | 25 | 27 |
| Ascomycota | 17 | 13 | 0 | 10 |
| Bacteroidetes | 9 | 8 | 5 | 3 |
| Chlorobi | 1 | 1 | 0 | 0 |
| Chloroflexi | 5 | 5 | 3 | 3 |
| Cyanobacteria | 12 | 11 | 5 | 4 |
| Deinococcus-Thermus | 1 | 1 | 1 | 1 |
| Firmicutes | 4 | 4 | 2 | 2 |
| Gemmatimonadetes | 1 | 1 | 1 | 1 |
| Planctomycetes | 5 | 5 | 5 | 5 |
| Proteobacteria | 56 | 58 | 36 | 33 |
| Streptophyta | 1 | 0 | 1 | 0 |
| unclassified (derived from Bacteria) | 1 | 1 | 0 | 1 |
| unclassified (derived from Viruses) | 1 | 1 | 1 | 1 |
| Verrucomicrobia | 4 | 4 | 3 | 2 |
| Grand Total | 158 | 154 | 92 | 97 |
